# The landscape of renal replacement therapy in Veterans Affairs Medical Center intensive care units

**DOI:** 10.1080/0886022X.2021.1949347

**Published:** 2021-07-14

**Authors:** Chandan Vangala, Maulin Shah, Natasha N. Dave, Layth Al Attar, Sankar D. Navaneethan, Venkat Ramanathan, Susan Crowley, Wolfgang C. Winkelmayer

**Affiliations:** a Baylor College of Medicine, Houston, TX, USA; b Michael E. DeBakey Veterans Affairs Medical Center, Houston, TX, USA; c Houston Center for Innovations in Quality, Effectiveness, and Safety (IQuESt), Houston, TX, USA; d Yale School of Medicine, New Haven, CT, USA; e Veterans Affairs Connecticut Healthcare System, West Haven, CT, USA

**Keywords:** Continuous renal replacement therapy, prolonged intermittent renal replacement therapy, CRRT, PIRRT, veterans affairs, intensive care unit

## Abstract

**Background:**

Outpatient dialysis is standardized with several evidence-based measures of adequacy and quality that providers aim to meet while providing treatment. By contrast, in the intensive care unit (ICU) there are different types of prolonged and continuous renal replacement therapies (PIRRT and CRRT, respectively) with varied strategies for addressing patient care and a dearth of nationally accepted quality parameters. To eventually describe appropriate quality measures for ICU-related renal replacement therapy (RRT), we first aimed to capture the variety and prevalence of basic strategies and equipment utilized in the ICUs of Veteran Affairs (VA) medical facilities with inpatient hemodialysis capabilities.

**Methods:**

*Via* email to the dialysis directors of all VA facilities that provided inpatient hemodialysis during 2018, we requested survey participation regarding aspects of RRT in VA ICUs. Questions centered around the mode of therapy, equipment, solutions, prescription authority, nursing, anticoagulation, antimicrobial dosing, and access.

**Results:**

Seventy-six centers completed the questionnaire, achieving a response rate of 87.4%. Fifty-five centers reported using PIRRT or CRRT in addition to intermittent hemodialysis. Of these centers, 42 reported being specifically CRRT-capable. Over half of respondents had the capabilities to perform PIRRT. Twelve centers (21.8%) were equipped to use slow low efficient dialysis (SLED) alone. Therapy was largely prescribed by nephrologists (94.4% of centers).

**Conclusions:**

Within the VA system, ICU-related RRT practice is quite varied. Variation in processes of care, prescription authority, nursing care coordination, medication management, and safety practices present opportunities for developing cross-cutting measures of quality of intensive care RRT that are agnostic of modality choice.

## Summary

The Veterans Health Administration is the largest integrated healthcare system in the United States. Using the Veterans Affairs (VA) internal Dialysis Facility Directory, we sent surveys regarding renal replacement therapy practices in the intensive care unit to the dialysis directors of each VA facility that provided VA-staffed inpatient hemodialysis. With 76 responses out of total of 87 facilities, we described prolonged and continuous renal replacement therapy with regards to basic aspects of treatment, such as modality, equipment, solutions, anticoagulation, staffing, and access. This report helps educate on the heterogeneity of treatment and what principal common denominators could serve as potential quality measures to improve the delivery of care.

## Background

Prolonged intermittent renal replacement therapy (PIRRT) and continuous renal replacement therapy (CRRT) are frequently utilized in the intensive care unit to provide a more sustained duration of solute and volume control in patients with tenuous hemodynamics. Outpatient renal replacement therapies, namely, intermittent hemodialysis (IHD) and peritoneal dialysis, have evidence-based measures that serve as guides to therapy [1]. While not hospitalized, patients are more likely to be in ‘steady state’, and nephrologists have goals for adequacy, phosphorus control, nutritional parameters, access, interdialytic weight gain, etc. However, in the intensive care unit, therapy remains varied by choice of modality, duration, and several ancillary factors relating to access, interruptions, and drug dosing.

The mortality of patients with acute kidney injury requiring IHD has improved in recent years [2]. However, patients with acute kidney injury requiring CRRT continue to face an estimated mortality rate of >60% [3–6]. This discrepancy suggests more dire pathology and perhaps, a role in improving the quality of care delivered with this therapy. The opportunity to delineate quality care may be best outlined within the largest integrated healthcare system in the United States, the Veterans Health Administration. Yet, a formidable obstacle in this goal is the facility-dependent variability surrounding prolonged and continuous renal replacement therapy.

Standardized goals or quality measures may be more easily identified with a basic understanding of how therapy is administered across the country. We aimed to understand the variety of strategies and equipment utilized. In doing so, we also hoped to highlight broadly applicable parameters of ICU care that are both agnostic to modality choice and relevant to all settings, VA and non-VA alike. Through an online survey, we conducted a descriptive analysis of RRT provided in VA-based ICUs.

## Methods

We emailed the dialysis directors of all 87 Veterans Affairs medical centers with VA-staffed inpatient hemodialysis capabilities identified in VA’s internal Dialysis Facility Directory and requested their voluntary participation in a questionnaire. We asked questions regarding general strategies and equipment for RRT in the ICU (Supplemental Figure F1). Dialysis directors were encouraged to answer the questionnaire or suggest alternate staff that would be best equipped to answer questions about ICU-related care. We obtained the VA station name from which a response was obtained; no other identifying information was collected.

**Figure 1.**
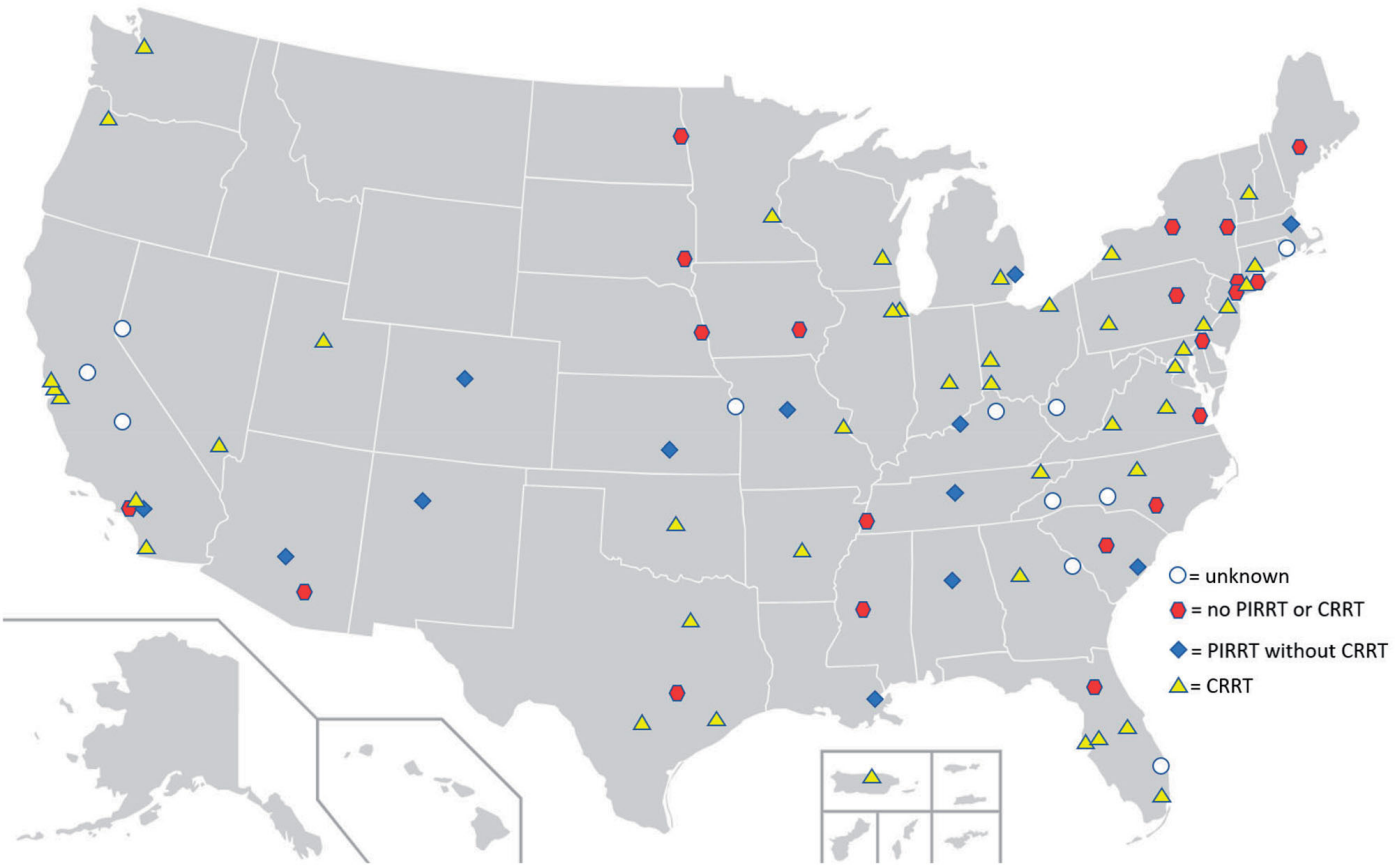
Map of types of renal replacement therapy used in veterans affairs intensive care units with intermittent hemodialysis capabilities. Accessed and edited from Wikimedia Commons with permission from the author.

The survey was conducted using an online platform, SurveyMonkey^®^ (www.surveymonkey.com), that utilized Secure Sockets Layer encryption. Our emailed request for participation included a URL, which directed responders to the online survey consisting of 21 questions (Supplemental Figure F1). The first question identified the VA institution where the responder was located. Fourteen of the questions were in the multiple-choice format while five questions were ‘yes/no’, and one question was an optional short answer allowing for a follow-up explanation of a previous answer. The questionnaire remained available to responders from 1 December 2018 to 15 March 2019. Responses were assessed as proportions. Only one response was allowed per participating center, but changes to answers were permitted at any time. Due to the acquisition of de-identified data, our request for IRB approval was waived (H-45304 memorandum) at the Baylor College of Medicine and Michael E. DeBakey VA, Houston, Texas, as it did not fall under the category of human subjects research.

## Results

Of the 87 institutions that were requested to participate in the survey, 76 centers responded. Of those 76 centers, 21 (27.6%) centers reported IHD as the available modality of RRT and did not utilize prolonged or continuous therapies in the ICU. We found that 55 (72.4%) reported employing PIRRT or CRRT as RRT options. Of these, 13 (17.1%) centers utilized PIRRT without being CRRT-capable while 42 (55.3%) centers reported being CRRT-capable. Fewer centers with inpatient HD capabilities were located in the Rocky Mountain or West North Central region, but no obvious geographic trend toward particular types of therapies was evident (see Figure 1). Thirty-four centers (44.7%) could provide inpatient peritoneal dialysis. A larger proportion of facilities with higher operational complexity were found to have CRRT capabilities (see Table 1).

**Table 1.** Renal replacement therapy by operational complexity.

Operational complexity	
Standard	*n* = 1
IHD	1 (100.0)
Intermediate	*n* = 13
IHD only	6 (46.2)
CRRT	6 (46.2)
PIRRT	5 (38.5)
PIRRT alone	1 (7.7)
SLED alone	1 (7.7)
Complex	*n* = 62
IHD only	14 (22.6)
CRRT	36 (58.1)
PIRRT	23 (37.1)
PIRRT alone	12 (19.4)
SLED alone	11 (17.7)

Among those that provided CRRT, respondents reported almost equivalent CVVH (54.5%) and CVVHD (56.4%) capabilities; slightly fewer centers provided CVVHDF (47.3%). Slow low efficient dialysis was the predominant mode of PIRRT. Five centers reported utilizing prolonged veno-venous hemodialysis, and one center employed this mode as their primary mode of therapy in the ICU. Only one center reported utilizing prolonged or accelerated veno-venous hemofiltration, and while that center could perform CVVHD, this mode of PIRRT was their primary form of RRT (see Table 2).

**Table 2.** Variety of prolonged and continuous renal replacement therapy.

Type of prolonged or continuous renal replacement therapy (*n* = 55)	Respondents, *n* = 55 *n* (%)
CRRT (CVVH/CVVHD/CVVHDF)	42 (76.4)
CVVH	30 (54.5)
CVVHD	31 (56.4)
CVVHDF	26 (47.3)
PIRRT	28 (50.9)
SLED	23 (41.8)
Other	6 (10.9)
Exclusively PIRRT	13 (23.6)
Exclusively SLED	12 (21.8)

CRRT: continuous renal replacement therapy; CVVH: continuous veno-venous hemofiltration; CVVHD: continuous veno-venous hemodialysis; CVVHDF: continuous veno-venous hemodiafiltration; PIRRT: prolonged intermittent renal replacement therapy; SLED: slow low efficient dialysis.

Twenty-seven percent of centers with capabilities for prolonged or continuous therapy would access a fistula or graft for therapy. All centers reported the requirement of having an HD nurse with constant visualization of the access as a special safety precaution for this practice, which is consistent with the VA National Center for Patient Safety’s recommended practice [7]. More than half of the facilities endorsed using triple-lumen, non-tunneled, temporary catheters equipped with an additional port for infusions. The catheter lengths utilized were highly varied, but the majority reported having catheters of lengths 15–16 and 19–20 cm (see Table 3).

**Table 3.** Access-related responses.

Access	*n* (%)
Anticoagulant locking (responses = 76/76; 100%)	
Temporary catheter	55 (72.4)
Tunneled dialysis catheter	52 (68.4)
Locking content (responses = 76/76; 100%)	
Citrate	9 (11.8)
Heparin	47 (61.8)
Saline	19 (25.0)
Alteplase	1 (1.3)
Catheter length (responses = 68/76; 89.5%)	
12–13.5 cm	21 (30.9)
15–16 cm	42 (61.8)
19–20 cm	53 (77.9)
23–25 cm	31 (45.6)
>25 cm	7 (10.3)
Temporary triple-lumen dialysis catheter use during renal replacement therapy (responses = 76/76; 100%)	41 (54.0)
Arteriovenous fistula/graft use during CRRT/PIRRT (responses = 55/55; 100%)	15 (27.3)

Prescribers of therapy were largely nephrologists, consistent with VHA policy [8], with only one center relying on intensivists for RRT. All centers had ICU nurses directly managing CRRT with 23.1% of centers having HD nurses facilitate set-up. By contrast, with PIRRT, the majority of therapy was managed by HD nurses, usually for slow low efficient dialysis (SLED). Nearly 63% of centers had either an ICU or HD nurse stationed one-to-one with patients requiring prolonged or continuous therapies (see Table 4).

**Table 4.** Staffing-related responses.

Providers of prolonged/continuous therapy (responses = 55/55)	*n* (%)
Intensivist	1 (1.8)
Nephrologist	52 (94.5)
Combined intensivist/nephrologist	2 (3.6)
Direct manager of CRRT (responses = 41/42; 95.2%)	
ICU nurse	31 (77.5)
HD nurse	1 (2.4)
HD nurse set-up, ICU manage	9 (23.1)
Direct manager of PIRRT (responses = 28/28; 100.0%)	
ICU nurse	3 (10.7)
HD nurse	17 (60.7)
HD nurse set-up, ICU manage	8 (28.6)
1:1 staffing (responses = 54/55; 98.2%)	34 (63.0)
1:1 for PIRRT only (responses = 8/13; 61.5%)	4 (50.0)
1:1 for CRRT only (responses = 26/27; 96.3%)	17 (65.4)

**Figure 2.**
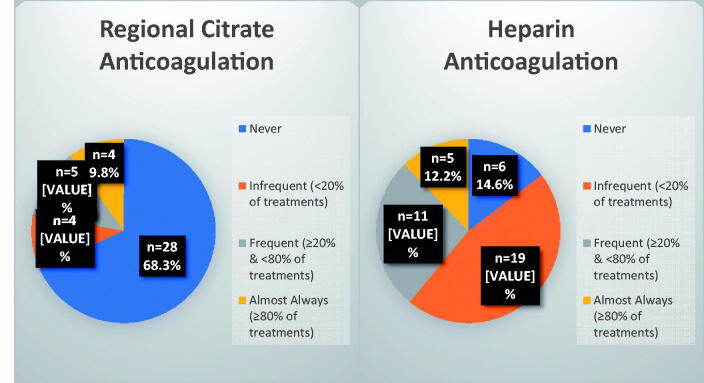
Anticoagulation on continuous renal replacement therapy.

Compared to regional citrate anticoagulation, heparin anticoagulation appeared to be utilized with more frequency during CRRT (see Figure 2). Among facilities that provided CRRT, most (68.3%) reported never using regional citrate anticoagulation. During SLED, 70% of respondents reported never using regional citrate anticoagulation and 30% reported infrequently (<20% of the time) using regional citrate anticoagulation. As with CRRT, heparin anticoagulation also remained slightly more prevalent during SLED (see Supplemental Table S2). Of the 40 centers that practiced CRRT, nearly two-thirds used one brand’s device and the other third used an alternate brand’s device (see Supplemental Table S1). One brand’s solutions appeared to be the most commonly adopted with 70.7% of centers endorsing use. One institution generated its own tailor-made replacement fluid. Of the CRRT-capable centers, 46.2% carried therapy fluid with both 2 and 4 mEq/L of potassium, while 25.6% carried therapy fluid of all three varieties of common potassium concentrations (see Supplemental Table S1). Antibiotic dosing was frequently a multidisciplinary practice, with 81.8% of centers relying on the input of pharmacists, and 20.8% of centers solely relying on pharmacist input. The addition of nephrology input to the pharmacy was present in 62.2% of centers. A multidisciplinary effort among the primary intensivist, nephrologist, and pharmacist was reported in 35.8% of centers (see Supplemental Table S3).

## Discussion

Among VA medical centers with capabilities for inpatient HD, the use of prolonged or continuous renal replacement therapy in the ICU is not uncommon. Of the 55 centers that offer PIRRT and/or CRRT, more than three-quarters reported some form of continuous therapy. While not directly assessed in this study, practitioner preference, facility-level constraints related to staffing, cost, and utility, available surgical services, and coordination with ICU staff are likely some of the determinants of therapy availability. Importantly, no definitive evidence regarding mortality or kidney recovery exists to support the use of one particular mode of kidney replacement therapy over another [9,10]. Continuous therapies offer a practical advantage of gradual solute and volume control, particularly useful in post-operative patients with acute kidney injury.

The above conveniences of CRRT come at the cost of additional staffing and equipment, both of which require training, vigilant monitoring, and management [11]. Accordingly, facilities with a higher level of operative complexity, and therefore more complex surgical services offered, were more likely to be equipped with CRRT. Flexible staffing presents a critical constraint that often determines therapy availability. CRRT is mostly set up and managed by ICU nurses while PIRRT, particularly SLED, appears to have more involvement of HD nurses. While not assessed in this study, this division and staffing availability from either department could influence the modes of therapy made available within a center.

One-to-one nurse staffing was employed among the majority of VA facilities that offered prolonged or continuous therapy. This staffing model supports the VA National Center for Patient Safety’s recommendation for continuous visual monitoring of dialysis vascular access, especially in patients at the highest risk for bleeding from access dislodgement, such as those receiving RRT in the ICU setting [12]. More thorough investigations of staffing models are needed to better understand responsiveness and ability to troubleshooting circuit failure and how well-prescribed therapies are delivered. As critical care complexity grows with extracorporeal membrane oxygenation and ventricular assist devices, nursing teams dedicated to RRT initiation and troubleshooting may become valuable [13].

More than half of VA facilities surveyed are PIRRT-capable. Comparative advantages relative to CRRT include less depletion of water-soluble minerals that are easily filtered [14] and reduced losses of amino acids and protein [15], potentially enabling the achievement of recommended net protein balance [16,17]. While PIRRT is understudied in this regard and dosing is highly variable, the extended periods without clearance could potentially result in fewer nutritional losses, which may be key to some patients’ recoveries. Moreover, the cost of staffing with one-to-one nursing time, the increased potential for clotting and blood loss, and reduced patient mobility all present additional setbacks to CRRT use. PIRRT fills a need for transitional therapy that limits many of the detrimental aspects of CRRT and the cost of additional staffing. However, as discussed later, drug dosing is not well-understood and may limit providers’ comfort with use in patients with critical needs for accurate dosing of medications within therapeutic indices. While no modality has demonstrated superiority in large generalizable trials, with further study there potentially exists an opportunity to outline select patients for whom PIRRT offers fewer complications.

Both the U.S. General Service Administration’s centralized procurement system and the choice of RRT may influence purchasing decisions among VA centers. Survey findings indicate that one brand’s therapy fluids are most commonly used, likely because its replacement fluid and dialysates are compatible with all forms of continuous therapy (CVVH, CVVHD, and CVVHDF) and with both CRRT devices used within the VA system (see Supplemental Table S1). The majority of VA institutions with CRRT capabilities utilize a single vendor’s device and therapy fluid. Therapy fluids content differs minimally across the three major manufacturers reported to be used by VA facilities, and therefore, is unlikely to yield distinct clinical outcomes per solution type.

Regarding catheter care, and specifically locking solutions, limited quality evidence exists to support the use of any particular packing solution over another, and heparin use has become quite common. Our study similarly reflects that heparin is the most commonly used locking agent among VA centers. Whether it presents advantages over saline remains uncertain, and yet it is the standard by which alternative anticoagulants are assessed [18]. In a Cochrane review of 27 studies conducted by Wang et al., tissue plasminogen activator was the only agent that reduced catheter malfunction over the standard of care [18]. However, the bleeding concern associated with injection or leak is a considerable deterrent, especially in surgical intensive care units. Notably, only one VA facility reported using a tissue plasminogen activator for locking catheters. While not uniformly consistent, some studies suggest a reduced incidence of catheter-related bacteremia with citrate use [18]. Importantly, most of these studies were not conducted in AKI requiring RRT, and the largest study to date to compare heparin *vs.* citrate use in patients with AKI requiring RRT *via* non-tunneled dialysis catheter noted no difference in event-free survival of catheter, thrombosis, infection, or adverse events [19]. Thus, currently, no uniform recommendation regarding catheter-locking can be suggested.

Slightly more than half of the respondents reported using triple-lumen temporary catheters. The impact of this additional port on catheter-related infection is unknown, but just as muddy is the potential for increased clearance of vital medicines, particularly antibiotics, infused through these ports. Interestingly, almost a third of respondents carried catheters of around 12–13.5 cm, which may have limited utility in a predominantly male VA population [20,21]. However, we did not assess how frequently these smaller catheters were deployed, and therefore, we cannot comment on the appropriateness of use.

Primary strategies for reducing blood loss from filter clotting include preemptive filter changes or anticoagulation. Anticoagulation has the added benefit of limiting time away from therapy, as changing out filters requires substantial disassembly and set-up. It also does not incur the costs of additional filters, but whether this is more cost-effective is still uncertain given the additional medications and monitoring required with anticoagulation. Compared to heparin, regional citrate anticoagulation in select patients has demonstrated increases in filter life [22] as well as decreases in complication rates, blood loss, and therapy interruptions [23–26]. The Kidney Disease: Improving Global Outcomes 2012 guideline on acute kidney injury maintains a 2B recommendation for anticoagulation use in patients requiring CRRT, but only in those who do not have an ‘increased bleeding risk or impaired coagulation’ or liver dysfunction or shock, and only in centers with an established protocol [27]. The use of regional citrate anticoagulation is limited to 0–20% of patient treatments whereas heparin remains the most common form of anticoagulation worldwide in CRRT [27]. Heparin use is likewise favored among VA centers with CRRT capabilities, with regional citrate anticoagulation practiced by ∼1/3rd of facilities (see Figure 2). The complexity of coordinating across several disciplines – physicians, pharmacy, nursing, respiratory therapy, laboratory – to manage two infusions with frequent monitoring may be the main aversion to use. Despite studies endorsing safety, the potential for error-induced hypocalcemia and citrate accumulation or net citrate overload may present significant concerns for centers with limited prior experience.

Antimicrobial dosing for patients on RRT in the ICU is immensely challenging, and practitioners in the United States frequently rely on dosing nomograms with limited supporting evidence. Variability among intrinsic patient characteristics, clinical condition, and preferred prescriptions by different practitioners render valid clearance studies in CRRT rather difficult; however, theoretical estimates of clearance can be made from the total delivered therapy fluid dose and the protein-binding of that drug [28]. Unfortunately, therapeutic drug monitoring for most antimicrobials other than vancomycin and aminoglycosides is not practically available in the United States, and real-world evidence for dosing is limited to studies using more antiquated methods of CRRT. More often than not, patients requiring CRRT are administered inadequate doses of antimicrobials [29–31]; similar concerns exist with SLED [32]. Limited guidance exists for other forms of PIRRT as the therapy results in both periods of substantial clearance and negligible clearance. The only available guidance for dosing antimicrobials on modes of PIRRT other than SLED is derived from in silica studies [33–35], limiting many providers’ comfort with use in patients with infections or substantial concerns for infections. Even in SLED, a particular form of PIRRT that has historically been in use longer than other modes, pharmacists do not agree on the dosing of commonly used antimicrobials [36]. Thus, sole reliance on pharmacy input may be insufficient for accurate dosing. The dynamic nature of patient courses and complementary therapy chosen necessitates frequent interdisciplinary communication regarding medication dosing. The majority of VA centers, as reported by nephrologists or their designees, endorse input from both pharmacy and nephrology regarding antimicrobial dosing. Mechanisms that ensure that providers who order antimicrobials are frequently (at least daily) educated of the renal replacement therapy plan are paramount to ensuring our best estimations of drug doses.

Limitations of this study include reliance on providers’ recall. We likely garnered reliable responses regarding modes of therapy, machine type, solution type, and other variables related to therapy that is commonly encountered daily. However, the actual use of more granular aspects of care, such as initiation and stopping points for RRT, frequency of PIRRT, nutritional support, diuretic use, deployment of the catheter of specific lengths (as compared to stock availability), and anatomical location of catheter insertions are not captured. Additionally, questions regarding frequency of anticoagulation and provider input on antimicrobial dosing are more subjective impressions of the respondents. Regardless, these questions still served the purpose of underscoring the preferential equipment and supply use, anticoagulation strategies, nurse staffing models, and multidisciplinary input into antimicrobial dosing for RRT within the VA. Unfortunately, comparable data outside the VA system was not available. Additionally, to keep the questionnaire brief enough to maximize the response rate, we were limited in how comprehensive our study could be. Other than noting the prevalence of peritoneal dialysis, we did not assess any characteristics or equipment related to peritoneal dialysis therapy. We did not request responses regarding the French size of catheters, or whether catheters were straight, pre-curved, or with curved extensions. We also did not capture adherence to other VA-required RRT practice standards (e.g., mandatory time-out pre-RRT, dedicated water hookups, adherence to current ISO water standards, completion of life-sustaining treatment directive). We did not capture whether the pharmacy assisted with therapy fluid preparation. Eleven centers with inpatient HD capabilities did not complete the survey. This study does not report on any specific clinical outcomes related to practice variation. Our use of a simpler online tool with less flexibility allowed for non-responses among CRRT-capable surveyees, an option intended to be reserved for respondents that were exclusively HD-capable. Nonetheless, with a response rate of 87.4% and only a few inappropriate non-responses among seven questions, we believe that we received a sizable sample that serves to adequately describe the variety of strategies and equipment surrounding RRTs in Veteran Affairs ICUs.

Within the largest integrated healthcare system in the United States, we described diverse approaches to RRT. While we documented significant variation in modality and supply use, this study uncovers significant opportunities to explore measures of quality care that are agnostic of RRT modality. Except for catheter size and adequate length per location, limited evidence exists to distinguish particular equipment or solutions. The broader, primary objective of continuous and prolonged renal replacement therapy is to deliver precise and consistent treatment that minimizes harm to patients. Aligned with this aim, practitioners should ideally deliver adequate and steady solute and volume control, while concomitantly limiting blood loss, nutritional deficiencies, and errors in drug dosing. Given the variety of treatments, broadly applicable quality measures should be an initial area of focus for improving CRRT and PIRRT. Previous suggestions include ratios of prescribed to delivered therapy, time devoted to malfunction-related suspension of therapy, assessments of target solute clearance in the effluent, filter clots, and blood loss [37]. As highlighted in this study, facility and organizational level variation exists in several other processes of care related to RRT in the ICU. By their RRT modality independence, parameters, such as care coordination with antimicrobial dosing, nursing models for RRT delivery, RRT prescription authority, vascular access insertion and management, institutional safety standards, may also be well-suited for the assessment of cross-cutting quality measures in the future. These broadly applicable parameters warrant further study and are not VA-specific, but rather relevant to RRT in non-VA facilities as well. The landscape of RRT in the ICU has never been described at this level, and this report serves to educate further on options for addressing ICU-related care.

## Supplementary Material

Supplemental Material

## Data Availability

All relevant data are included in the paper.

## Abbreviations

CVVH: continuous veno-venous hemofiltration
CVVHD: continuous veno-venous hemodialysis
CVVHDF: continuous veno-venous hemodiafiltration
CRRT: continuous renal replacement therapy
ICU: intensive care unit
IHD: intermittent hemodialysis
PIRRT: prolonged intermittent renal replacement therapy
RRT: renal replacement therapy
SLED: slow low efficiency dialysis
VA: veterans affairs
