# Supplementary material for: The landscape of renal replacement therapy in Veterans Affairs Medical Center intensive care units
Source: Ren Fail. 2021 Jul 14;43(1):1146–54. doi: 10.1080/0886022X.2021.1949347 (PMC8280999; doi:10.1080/0886022X.2021.1949347)
Supplement: Supplemental Material [file IRNF_A_1949347_SM0982.pdf]

Supplemental Figure F1: Questionnaire Assessing Renal Replacement Therapy Equipment and Strategy in the Intensive Care Unit

Our aim is to conduct a study that describes the modalities and equipment used for renal replacement therapy in Veterans Affairs intensive care units. Your participation in this survey is voluntary, and by completing this survey your consent is implied. Thank you for participating, and we appreciate your time.

1. At which Veteran Affairs medical center do you practice?

____

2. Please select all of the intensive care units that exist at your facility.

□ Medical Intensive Care Unit

□ Cardiac Care Unit

□ Surgical Intensive Care Unit

□ Not Applicable (ICU care not available)

□ Other (please specify): _________________

3. What modes of renal replacement therapy do you offer in the ICU? Please check all that apply.

□ Peritoneal Dialysis

□ Intermittent Hemodialysis

□ Continuous Veno-venous Hemofiltration (CVVH)

□ Continuous Veno-venous Hemodialysis (CVVHD)

□ Continuous Veno-venous Hemodiafiltration (CVVHDF)

□ Slow Low Efficient Daily Dialysis (SLED)

□ SHIFT (PIRRT)

□ Accelerated Veno-venous Hemofiltration (PIRRT)

□ Other: ______________________

4. Does your institution pack temporary dialysis catheters with an anticoagulant in the intensive care unit?

□ Yes

□ No

5. Does your institution pack tunneled dialysis catheters with an anticoagulant in the intensive care unit?

□ Yes

□ No

6. What are the catheters packed with in between use in the intensive care unit?

□ Citrate

□ Heparin

□ Saline

□ Not applicable

□ Other: ___________

7. Does your institution utilize trialysis catheters (short-term dialysis catheter with an additional injectable third lumen for administration of medications)?

□ Yes

□ No

8. Please select all of the temporary catheter lengths that most closely resemble what your institution carries.

□ 12-13.5cm

□ 15-16cm

□ 19-20cm

□ 23-25cm

□ >25cm

Other: ___________

9. Please identify who orders prescriptions for prolonged or continuous therapies (CVVH, CVVHD, CVVHDF, SLED, SHIFT, AVVH)

□ Intensivist alone

□ Nephrologist alone

□ Combined intensivist/nephrologist

□ Not Applicable

□ Other: ________________________

10. Please identify the staff member who directly manages renal replacement therapy if a patient is on a continuous therapy (CVVH, CVVHD, CVVHDF):

□ Intensive Care Unit nurse alone

□ Hemodialysis nurse alone

□ Hemodialysis nurse set- up/oversight and Intensive Care Unit nurse management

□ Not applicable

□ Other: _______________________

11. Please identify the staff member who directly manages renal replacement therapy if a patient is on a prolonged therapy that is not continuous (SHIFT, AVVH, SLED):

□ Intensive Care Unit nurse alone

□ Hemodialysis nurse alone

□ Hemodialysis nurse set- up/oversight and Intensive Care Unit nurse management

□ Not applicable

□ Other: _______________________

12. If an ICU nurse is taking care of a patient on a continuous or prolonged therapy (CVVH, CVVHD, CVVHDF, SLED, SHIFT, AVVH), does that nurse only take care of one patient? In other words, are they stationed 1:1?

□ Yes

□ No

□ Not Applicable

□ Other: __________________________

13. Does your hospital use grafts or fistulas during any prolonged or continuous therapy (CVVH, CVVHD, CVVHDF, SLED, PIRRT)?

□ Yes:

□ No

□ Not Applicable

□ Other: __________________________

14. If so, during which continuous or prolonged therapies (CVVH, CVVHD, CVVHDF, SLED, PIRRT) do you allow fistula or graft use? Please select all that apply

□ Continuous Veno-venous Hemofiltration (CVVH)

□ Continuous Veno-venous Hemodialysis (CVVHD)

□ Continuous Veno-venous Hemadiafiltration (CVVHDF)

□ Slow Low Efficient Daily Dialysis (SLED)

□ SHIFT (PIRRT)

□ Accelerated Veno-venous Hemofiltration (PIRRT)

□ Not Applicable

□ Other: ______________________

15. If so, please elaborate on any techniques or safeguards in place for graft/fistula use. Please type 'N/A' if you do not use grafts/fistulas during prolonged or continuous therapies.

____

16. Does your hospital use heparin anticoagulation with continuous or prolonged therapies (CVVH, CVVHD, CVVHDF, SLED, PIRRT)? Please estimate frequency of use:

□ Almost always (>80% of treatments)

□ Frequently (between 20-80% of treatments)

□ Infrequently (<20% of treatments)

□ Never (0% of treatments)

□ Not Applicable

17. Does your institution use regional citrate anticoagulation with continuous or prolonged therapies (CVVH, CVVHD, CVVHDF, SLED, PIRRT)? Please estimate frequency of use:

□ Almost always (>80% of treatments)

□ Frequently (between 20-80% of treatments)

□ Infrequently (<20% of treatments)

□ Never (0% of treatments)

□ Not Applicable

18. Once a patient is on continuous or prolonged therapy, who recommends adjustments to antimicrobial dosing? Please select all that apply

□ Pharmacist

□ Intensivist

□ Nephrologist

□ Not Applicable

19. For continuous or prolonged therapies (CVVH, CVVHD, CVVHDF, PIRRT) other than SLED, what machine do you use? Please select all that apply.

□ Prismaflex

□ NxStage

□ Not Applicable

□ Other: ___________

20. For continuous or prolonged therapies (CVVH, CVVHD, CVVHDF, PIRRT) other than SLED, what solutions do you use? Pleaser select all that apply

□ B. Braun/Duosol

□ NxStage

□ Baxter/Prisma

□ Not Applicable

□ Other: ________

21. For continuous or prolonged therapies (CVVH, CVVHD, CVVHDF, PIRRT) other than SLED, what potassium concentration solutions do you carry? Please select all that apply

□ 0k

□ 2k

□ 4k

□ Not Applicable

Supplemental Table S1: Equipment

**Machines Used for CRRT (responses=40; 95.2%)**	*n* (%)
PRISMAFLEX®	25 (62.5)
NxStage®	15 (37.5)
**Machines Used for PIRRT (responses=13/13; 100%)**	
PRISMAFLEX®	0 (0.0)
NxStage®	1 (7.7)
Other (SLED)	12 (92.3)
**Solutions used for CRRT (responses=41/42; 97.6%)**	
B Braun®/Duosol®	4 (9.8)
Baxter®	29 (70.7)
NxStage®	11 (26.8)
Tailor-made replacement fluid	1 (2.4)
**Potassium Solutions used for CRRT (responses=39/42; 92.9%)**	
0K	3 (7.7)
2K	1 (2.6)
4K	5 (12.8)
0K & 2K	0 (0.0)
0K & 4K	2 (5.1)
2K & 4K	18 (46.2)
0K, 2K, & 4K	10 (25.6)

Supplemental Table S2: Anticoagulation on Slow Low Efficient Dialysis

**Heparin Anticoagulation among SLED alone (responses=11/12; 91.7%)**	*n* (%)
Never	2 (18.2)
Infrequent (<20% of treatments)	5 (45.5)
Frequent (≥20% & <80% of treatments)	2 (18.2)
Almost Always (≥80% of treatments)	2 (18.2)
**Regional Citrate Anticoagulation among SLED (responses=10/12; 83.3%)**	
Never	7 (70.0)
Infrequent (<20% of treatments)	3 (30.0)
Frequent (≥20% & <80% of treatments)	0 (0.0)
Almost Always (≥80% of treatments)	0 (0.0)

Supplemental Table S3: Antimicrobial Dosing on Prolonged or Continuous Renal Replacement Therapy

**Antimicrobial Dosing (responses=53/55; 96.4%)**	*n* (%)
Pharmacist	11 (20.8)
Nephrologist	3 (5.7)
Intensivist	2 (3.8)
Pharmacist & Nephrologist	14 (26.4)
Pharmacist & Intensivist	1 (1.9)
Intensivist & Nephrologist	3 (5.7)
Pharmacist & Intensivist & Nephrologist	19 (35.8)
